# A Case Series and Literature Review of Angiosarcoma With Malignant Effusion—A Challenging Cytologic Diagnosis With Dire Prognostic Implications

**DOI:** 10.1002/dc.25471

**Published:** 2025-03-28

**Authors:** Jamie C. Y. Lam, Iris Y. H. Liu, Joanna K. M. Ng, Joshua J. X. Li

**Affiliations:** ^1^ Department of Pathology Queen Mary Hospital, the University of Hong Kong Hong Kong

**Keywords:** angiosarcoma, exfoliative cytology, fluid cytology, serous effusion

## Abstract

Angiosarcoma with malignant effusion is an uncommon yet clinically aggressive presentation, which poses as a diagnostic pitfall with its overlapping cytomorphologic features with metastatic adenocarcinoma. In this article, two cases reported in the literature were reviewed for cytology, immunocytochemistry, and clinical course. Key clinical hints, useful cytological features, and immunocytochemical markers in the diagnostic approach of this entity are presented.

## 1 Introduction

Diagnosis of malignant effusion is dependent on cytologic assessment. A broad range of causes underlie malignant effusion [1], and the plethora of possible diagnoses share multiple overlapping cytologic features. Non‐carcinomatous neoplasms, including lymphomas, melanomas, and sarcomas, are less frequently encountered differentials, which pose diagnostic challenges. In the case of sarcomas, specific cytomorphologic features may be lacking [2]. This is particularly problematic for angiosarcomas, as vasoformation can be mistaken for glandular differentiation and thus misinterpreted as adenocarcinomas [3, 4]. Such an issue is amplified by the frequent lack of correlation and examination of the primary tumor site in metastatic effusions and the differences in cytomorphology in exfoliative cytology compared to direct fine‐needle aspiration or tissue biopsy of primary lesions [5]. In this article, we present two cases of angiosarcoma with malignant effusion with a review of such cases available in the literature, aiming to detail the cytology, immunocytochemistry, and clinical course of angiosarcoma with malignant effusion for recognition of this uncommon but diagnostically significant entity.

### 1.1 Case Report

#### 1.1.1 Case 1

A 52‐year‐old woman with a history of nasopharyngeal carcinoma, treated with chemotherapy and radiotherapy presented with bloody sputum. Positron emission tomography–computed tomography revealed an extensive tumor involving the oropharynx and larynx with suspected metastatic lung lesions. Biopsy confirmed the diagnosis of angiosarcoma, and the patient started palliative chemotherapy (taxotere and cyclophosphamide). The patient completed four cycles of chemotherapy with radiological response. However, the patient developed left‐sided pleural effusion with desaturation. Drainage was performed and the pleural fluid was sent for cytologic examination. The patient deteriorated and succumbed 8 months after presentation.

**FIGURE 1:**
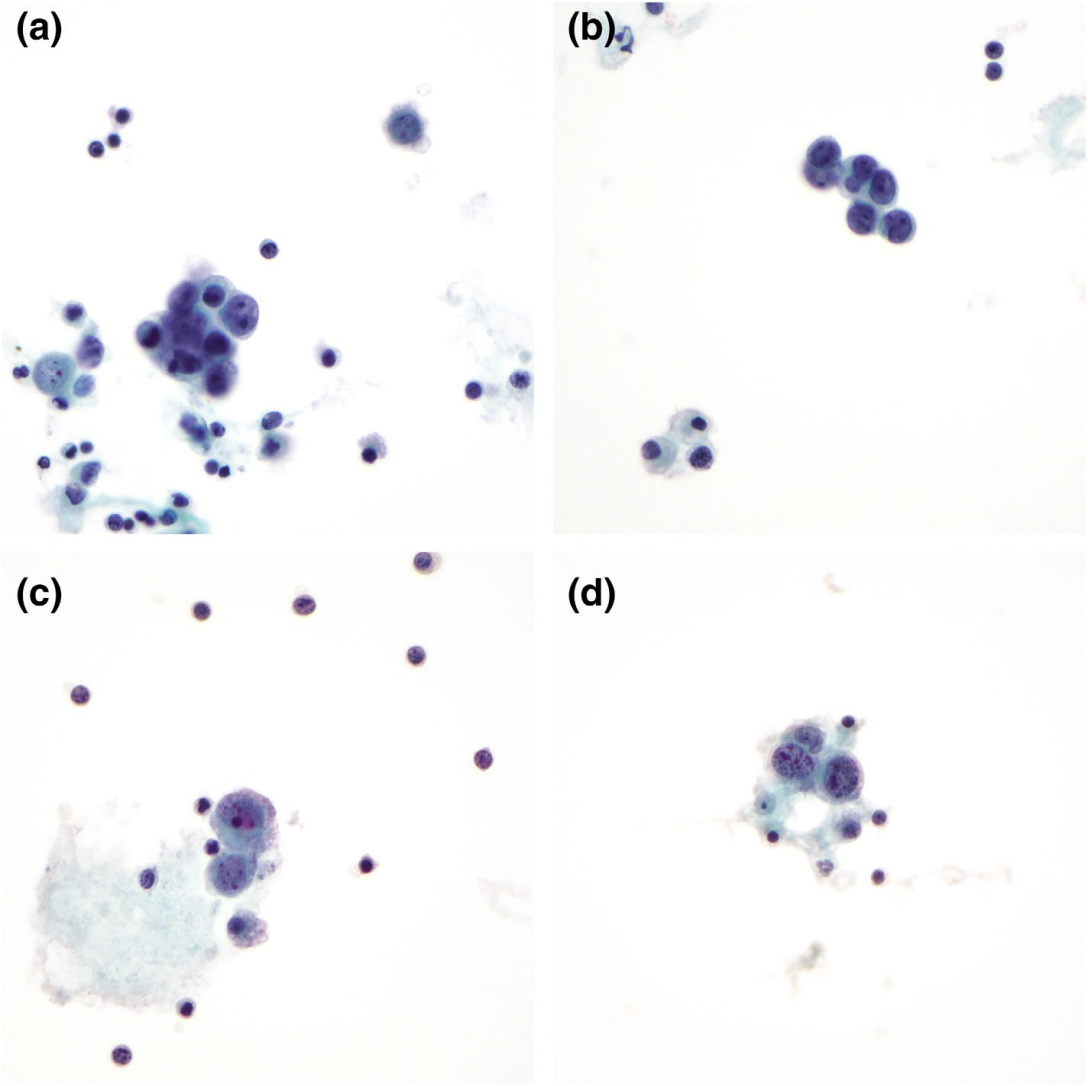
Case 1. (a) Small clusters of ovoid malignant cells, pap stain, 400× magnification. (b) Irregular nuclei with kidney‐bean‐like appearance, pap stain, 400× magnification. (c) Single to multiple nucleoli and macronucleoli, pap stain, 400× magnification. (d) Malignant cells in close association with polymorphs and suggestion of lumen formation, pap stain, 400× magnification.

**FIGURE 2:**
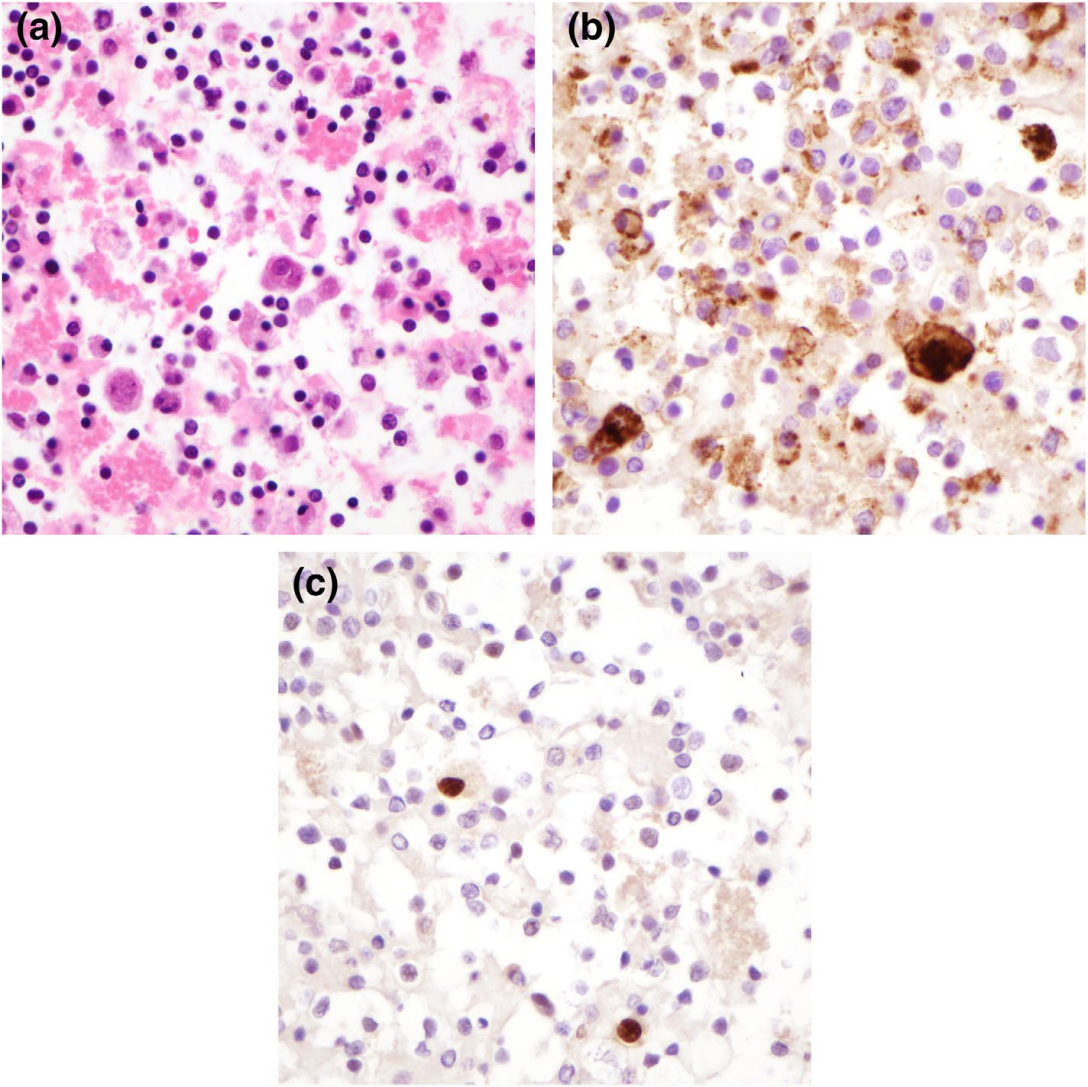
Cell block (agar block method) and immunocytochemistry. (a) Moderately cellular cell block preparation with malignant cells in a background of inflammatory cells, 400× magnification. (b) CD31, 400× magnification. (c) ERG, 400× magnification.

Cytospin preparation is of moderate cellularity and showed clusters of malignant cells in mostly small groups with ovoid outline (Figure 1a). The malignant cells demonstrated marked nuclear enlargement and irregularity with kidney‐bean‐like appearance (Figure 1b). Single to multiple nucleoli, including macronucleoli, were noted (Figure 1c). In the background were mononuclear cells and polymorphs with some clusters of malignant cells closely associated with polymorphs, suggestive of vascular lumen formation (Figure 1d). A cell block was prepared, and the malignant cells were immunoreactive to vascular markers (CD31 and ERG) (Figure 2).

#### 1.1.2 Case 2

A 94‐year‐old man presented with a right forehead mass for 6 months rapidly increasing in size, and with contact bleeding. He was seen by a dermatologist with high clinical suspicion of angiosarcoma. Initial skin biopsy from the forehead was non‐informative and before a repeat biopsy was performed, the patient developed left‐sided pneumothorax. Chest drain was inserted with pleural fluid sent for cytologic examination. Subsequent biopsy confirmed the diagnosis of angiosarcoma. Magnetic resonance imaging showed extensive tumor extension with orbital involvement. The patient deteriorated and succumbed 1 month after presentation.

**FIGURE 3:**
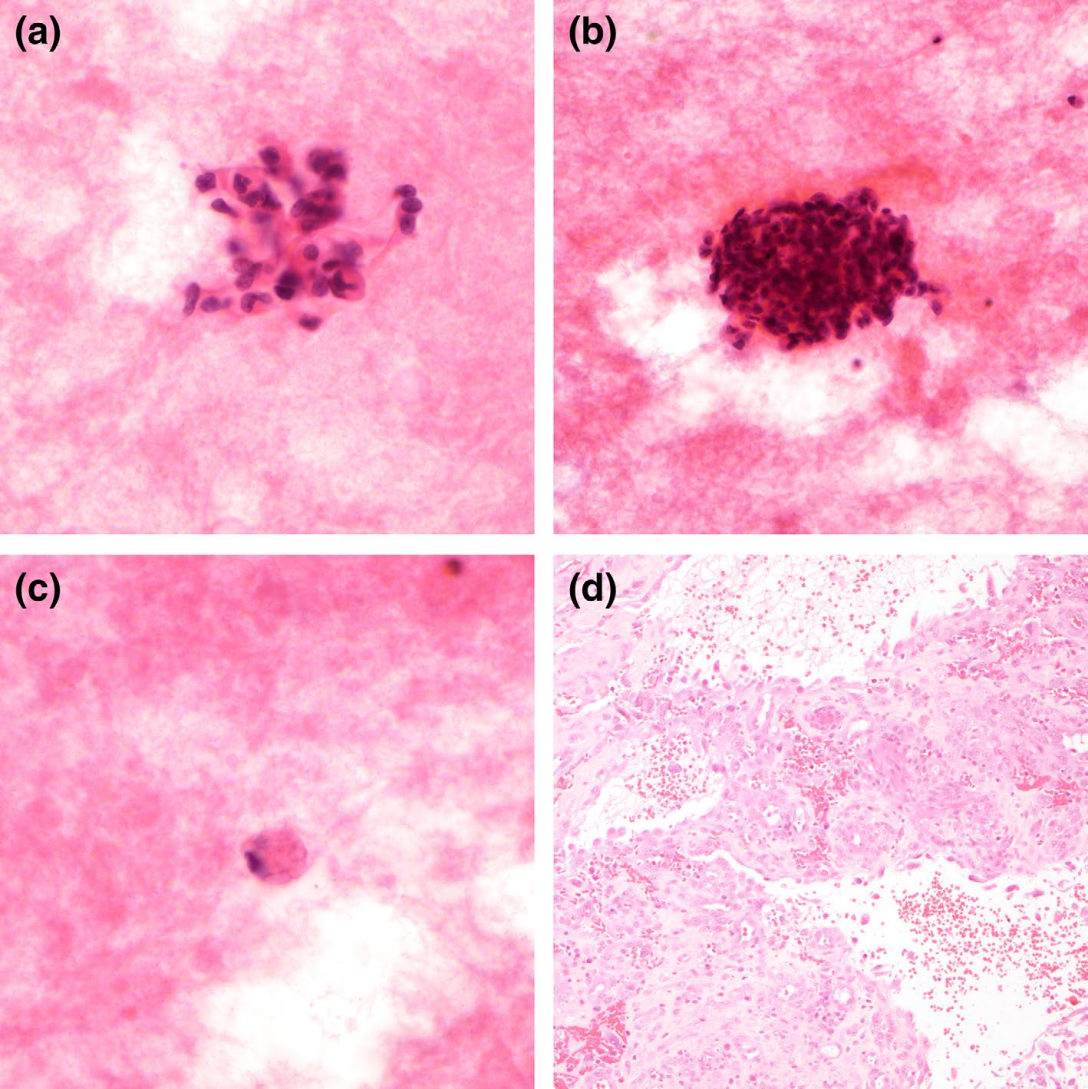
Case 2. (a) Spindled malignant cells with markedly irregular folded and indented nuclei, H&E, 400× magnification. (b) Mitotic and apoptotic figures, H&E, 400× magnification. (c) Intracytoplasmic multivacuolation, H&E, 400× magnification. (d) Corresponding tissue biopsy, 100× magnification.

On the smears, small clusters and singly spindled malignant cells were seen. The malignant cells displayed marked nuclear hyperchromasia and irregularity with nuclear folding and indentation (Figure 3a). Mitotic and apoptotic figures were noted (Figure 3b). Rare malignant cells with intracytoplasmic multivacuolation were noted (Figure 3c). These nuclear and cytoplasmic features corresponded with that of the issue biopsy (Figure 3d).

### 1.2 Literature Review

Literature search using the medical subject headings (MeSH) “angiosarcoma,” “pleural effusion,” “peritoneal effusion,” and “cytology” yielded 16 relevant articles [3, 4, 6, 7, 8, 9, 10, 11, 12, 13, 14, 15, 16, 17, 18, 19] describing 21 cases of angiosarcoma with malignant effusion. There were 5 cases of primary cardiac angiosarcoma, 3 epithelioid angiosarcomas, 1 radiation‐associated angiosarcoma, and 12 angiosarcomas not otherwise specified involving the pleural cavity only in 12 cases, and the pericardial cavity in 8. There was one case involving the peritoneal cavity, which also involved the pleural cavity. Of the 15 cases with follow‐up data, only one patient was alive with disease at 9 months; the remaining patients all died of disease, with the longest survival of 13 months (Table 1).

**TABLE 1:** Clinical characteristics of angiosarcoma with malignant effusion.

	No	Age	Sex	Diagnosis	Primary	Effusion cavity	Effusion on presentation	Treatment	Outcome	Cytologic diagnosis
Current series	1	52	F	Radiation‐associated angiosarcoma	Oropharynx/larynx	Pleural	No (5 months after diagnosis)	Chemotherapy (taxotere and cyclophosphamide)	DOD at 8 months	C5
	2	94	M	Angiosarcoma	Scalp	Pleural	No (during investigation)	Chest drain and pleurodesis	DOD at 1 month	C3
Dörr‐Jerat et al. [6]	3	73	F	Epithelioid angiosarcoma	Unknown	Pleural	Yes	Chest drain	Not mentioned	C2
Li et al. [7]	4	53	F	Primary cardiac angiosarcoma	Heart	Pericardial	Yes	Pericardial window	DOD at 6 months	C5
Ogino et al. [8]	5	67	M	Radiation‐associated angiosarcoma	Unknown	Pleural	Yes (8 years after radiotherapy)	Chemotherapy	DOD	C5
Kiwaki et al. [9]	6	60	M	Primary cardiac angiosarcoma	Unknown	Pericardial	Yes	Defaulted	DOD at 13 months	C2
Senthil Kumaran et al. [10]	7	41	M	Angiosarcoma	Unknown	Pericardial	Yes	Chemotherapy (paclitaxel)	DOD at 2 months	C3
Sharma et al. [4]	8	66	M	Angiosarcoma	Scalp	Pleural	No (11 months after diagnosis)	Chemotherapy and radiotherapy	DOD	C5
	9	82	M	Angiosarcoma	Acetabulum	Pleural	Yes	Radiotherapy	DOD	C5
	10	73	F	Angiosarcoma	Shin	Pleural	No (7 months after diagnosis)	Resection and radiotherapy	DOD	C5
	11	81	F	Angiosarcoma	Breast	Pleural + peritoneal	No (23 months after diagnosis)	Mastectomy and chemotherapy	DOD	C5
	12	77	F	Angiosarcoma	Breast	Pleural	No (11 months after diagnosis)	Mastectomy and chemotherapy	DOD	C5^a^
	13	73	M	Angiosarcoma	Axilla	Pleural	No (2 months after diagnosis)	Amputation	Lost to follow‐up	C5
Burns et al. [11]	14	23	F	Primary cardiac angiosarcoma	Heart	Pericardial	Yes	Pericardial window and chemotherapy (adriamycin + ifosfamide, then gemcitabine + docetaxel)	AWD at 9 months	C2
Durani et al. [12]	15	62	M	Epithelioid angiosarcoma	Unknown	Pleural	Yes	Thoracentesis	Not mentioned	C2
Geller et al. [13]	16	46	M	Angiosarcoma	Not mentioned	Pleural	Not mentioned	Not mentioned	Not mentioned	C5
Chen et al. [14]	17	69	M	Angiosarcoma	Unknown	Pleural	Yes	Thoracentesis	DOD at 1 month	C2
Riles et al. [15]	18	33	M	Angiosarcoma	Unknown	Pericardial	Yes	Surgical exploration	DOD at 1 month	C2
El‐Osta et al. [16]	19	64	M	Primary cardiac angiosarcoma	Heart (right atrium)	Pericardial	Yes	Resection and adjuvant chemotherapy (paclitaxel)	DOD at 5 months	C1
Saqi et al. [17]	20	41	M	Primary cardiac angiosarcoma	Heart (right atrium)	Pericardial	Yes	Not mentioned	Not mentioned	C5
Boucher et al. [3]	21	50	F	Epithelioid angiosarcoma	Not mentioned	Pleural	Yes	Not mentioned	Not mentioned	C4^a^
Randall et al. [18]	22	52	M	Angiosarcoma	Unknown	Pericardial	Yes	Pericardiocentesis	DOD at 2 months	C2
Ott et al. [19]	23	60	M	Angiosarcoma	Lung	Pleural	Yes	Resection	DOD at 2 months	C1

^a^ Cases 12 and 21 were misinterpreted as non–small cell carcinoma and suspicious for adenocarcinoma, respectively.

Approximately half (*n* = 10/21, 48%) were cytologically diagnosed as malignant (C5), with the remaining consisting of one suspicious (C4), one atypical (C3), seven negative (C2), and two nondiagnostic (C1) diagnoses. Two cases were misinterpreted as non–small cell carcinoma and suspicious for adenocarcinoma [3, 4]. Detailed cytologic descriptions were available in five articles. The lesional cells were discohesive or poorly cohesive, arranged in small clusters or single cells. Frequently reported cytomorphological features include marked nuclear irregularity and single to multiple prominent nucleoli. Vasoformative features were described in two articles, including hemophagocytosis, lumina, and vacuole formation. Another notable feature noted by Boucher et al. was the presence of a bloody background [3]. Positive immunocytochemical markers described were CD31, CD34, D2‐40, desmin, ERG, and factor VIII (Table 2).

**TABLE 2:** Cytomorphologic and immunocytochemical features of angiosarcoma in effusion fluid cytology.

Source	Cytomorphology	Other cytologic features	Immunocytochemistry
Current series	Single or small clusters of cells with marked nuclear irregularity, including nuclear folding, indentation and kidney‐bean‐like appearance, with nuclear enlargement and hyperchromasia	Vasoformative features—intracytoplasmic multivacuolation, lumina formation and close association with polymorphs	CD31+, ERG+; BerEp4−, calretinin−
Li et al. [7]	Large discohesive cells with enlarged nuclei, severe nuclear membrane irregularity and nuclear pleomorphism and macronucleoli		CD31+, D2‐40+, desmin+, ERG+, factor VIII+; AE1/3−, CD45−, heppar1−, MOC31−, myogenin−, SALL4−
Ogino et al. [8]	Aggregates of cells with eccentric nuclei and prominent nucleoli		
Geller et al. [13]^a^	Single or three‐dimensional clusters, multiple prominent or bar‐shaped nucleoli and chromatin strands, and abnormal mitoses	Vasoformative features—hemophagocytosis, cytoplasmic lumina/vacuoles containing red blood cells/neutrophils and endothelial wrapping	
Saqi et al. [17]	Small clusters of cells with scalloped borders Single pleomorphic epithelial‐appearing round cells with nuclei two to three times the size of the small lymphocytes, high nuclear‐to‐cytoplasmic ratios, round vesicular nuclei, and prominent nucleoli Isolated multinucleate tumor giant cells	Vasoformative features—erythron(hemo)phagocytosis and intracytoplasmic vacuoles	CD31+, CD34+ (focal); cytokeratin−, desmin−, EMA−, HHF‐35− (in corresponding tissue biopsy)
Boucher et al. [3]	Loosely cohesive groups to single epithelioid, polygonal cells, with hyperchromasia, irregularly distributed coarse chromatin, single to multiple nuclei that are oval to round with marked pleomorphism, high nuclear–cytoplasmic ratio, and conspicuous eosinophilic macronucleoli	Low cellularity with bloody background	CD31+; cytokeratin− (in corresponding tissue biopsy)

^a^ Described features in a series also include direct fine‐needle aspiration cytology of lesions.

## 2 Discussion

The incidence of sarcomas in leading to malignant effusion is significantly lower than carcinomas [20, 21]. Carcinomas with malignant effusions are often associated with dismal outcomes [22]. As for sarcomas, except for rare cases with radical treatment [23], involvement of serous cavities is unlikely to be compatible with long‐term survival [14, 15]. Compared to other sarcomas, angiosarcomas have a higher propensity for causing malignant effusions. Pericardial involvement in primary cardiac angiosarcomas is common, and the high risk of tumor bleeding and lung metastasis in angiosarcomas results in the classical presentation of bloody pleural effusion (hemothorax) [8, 21, 24]. As such, it is clinically relevant in recognizing such a presentation for making an accurate cytologic diagnosis.

As described in the current case series, angiosarcoma displays alarming cytomorphological features. Marked nuclear irregularity and single to multiple macronucleoli are consistent features noted by multiple authors [3, 7, 8, 13, 17]. In addition to the cytomorphologic features reported in the literature, mitotic and apoptotic figures were noted in Case 2 (Figure 3b), which can serve as a clue to the malignant behavior of the specimen. Vasoformation poses a diagnostic pitfall, as lumen formation and vacuolation can be mistaken for glandular formation, which resulted in misdiagnosis in two reported cases [3, 4]. Distinguishing features of angiosarcoma from adenocarcinoma are the lack of mucin production and a bloody background [3].

Additional literature on the cytomorphology of angiosarcomas on aspiration cytology, from excluded articles in the literature search and review of article references, was qualitatively reviewed. Two additional features were described in aspirates but not in effusion fluids, namely intracytoplasmic hemosiderin deposits and acinar‐like vascular structures [25, 26]. The lack of reports on such features in effusion cytology may be due to the small number of published cases that included cytomorphologic descriptions and that needle biopsy and aspiration may dislodge larger tissue fragments than passive tumor exfoliation [5].

Immunocytochemistry can be used to identify the singly malignant cells and to confirm vascular differentiation; useful markers include CD31, CD34, D2‐40, ERG, and factor VIII. At the same time, pertinent differentials should also be considered, and appropriate immunocytochemistry should be performed to exclude those diagnoses. The presence of prominent nucleoli and discohesiveness can suggest melanoma, while vacuolation can be seen in adenocarcinomas and macrophages. Supplementing vascular markers with macrophagic markers, melanoma markers, and cytokeratin [27] are good practices.

Specific clinical clues may be looked for when metastatic angiosarcoma is suspected. As in the current series, skin lesions and a history of radiotherapy were indicators of cutaneous angiosarcoma and radiation‐associated angiosarcoma, respectively. Primary cardiac sarcoma presents uniquely with heart failure symptoms, tamponade, and a cardiac mass. The progression of the primary lesion, metastatic deposits, and overall condition of the patient is rapid, and it is important to make a swift cytologic diagnosis to avoid treatment delay.

## 3 Conclusion

Angiosarcoma is distinctly associated with malignant effusion due to pericardial involvement in primary cardiac angiosarcoma and its bleeding tendency that often results in bloody effusion. Angiosarcoma can be recognized cytologically by its marked nuclear irregularity and single to multiple macronucleoli. However, features of vasoformation, lumen formation, and vacuolation overlap with adenocarcinomas and not uncommonly lead to misinterpretation. The lack of cohesiveness and a bloody background are hints differentiating these two overlapping entities. Clinical presentation and immunocytochemistry can contribute to achieving a correct diagnosis. The significance of recognizing this presentation lies in the quick disease tempo of metastatic angiosarcoma, and a quick cytologic diagnosis can ensure timely treatment.

## Author Contributions

**Jamie C. Y. Lam:** data curation, investigation, visualization, writing – original draft. **Iris Y. H. Liu:** data curation, investigation, visualization, writing – original draft. **Joanna K. M. Ng:** investigation, resources, validation. **Joshua J. X. Li:** conceptualization, investigation, methodology, supervision, visualization, writing – review and editing.

## Ethics Statement

The study was approved by the University of Hong Kong/Hospital Authority Hong Kong West institutional review board (reference number: 24–292).

## Consent

The study was granted the exemption of requiring written informed consent by the University of Hong Kong/Hospital Authority Hong Kong West institutional review board.

## Conflicts of Interest

The authors declare no conflicts of interest.

## Data Availability

All data generated or analyzed during this study are included in this article. Further enquiries can be directed to the corresponding author.
